# Effects of Wheat Malt Extract on Molecular and Behavioral Markers in Aged APP/PS1 and Wild-Type Mice

**DOI:** 10.3390/ijms27114994

**Published:** 2026-05-30

**Authors:** Aliya Kassenova, Evgeniy Svirin, Kseniia Sitdikova, Kirill Chaprov, Andrey Tsoy, Johannes de Munter, Anuar Nurzhanov, Maria Kuznetsova, Tatyana Veremeyko, Alexey Deykin, Eugene Ponomarev, Tatyana Strekalova, Sholpan Askarova

**Affiliations:** 1 Center for Life Sciences, National Laboratory Astana, Nazarbayev University, 010000 Astana, Kazakhstan; aliya.kassenova@nu.edu.kz (A.K.);; 2 Department of General Biology and Genomics, Faculty of Natural Sciences, L. N. Gumilyov Eurasian National University, 010000 Astana, Kazakhstan; 3 Center for Neurocognitive Research (MEG-Center), Moscow State University of Psychology and Education, 123290 Moscow, Russia; 4 Institute of Physiologically Active Compounds at Federal Research Center of Problems of Chemical Physics and Medicinal Chemistry, Russian Academy of Sciences, 119071 Chernogolovka, Russia; 5 Neuroplast B.V., 6222 NK Maastricht, The Netherlands; 6 Department of Biology, School of Sciences and Humanities, Nazarbayev University, 010000 Astana, Kazakhstan; 7 Department of Normal Physiology, Sechenov First Moscow State Medical University, 119991 Moscow, Russia; 8 Laboratory of Genetic Technology and Gene Editing for Biomedicine and Veterinary, National Research Belgorod State University, 308015 Belgorod, Russia; 9 Department of Biomedical Sciences, College of Biomedicine, City University of Hong Kong, Kowloon, Hong Kong; 10 Department of Biological Sciences and Bioinformatics, Xi’an Jiaotong-Liverpool University, Suzhou 215123, China; 11 Department of Psychiatry and Neuropsychology, Maastricht University, Universiteitsinsel 50, 6229 ER Maastricht, The Netherlands

**Keywords:** Alzheimer’s disease, APPswe/PS1E9 mice, wheat malt extract, wheat germ agglutinin

## Abstract

Growing evidence suggests an important pathogenetic role of brain-specific gangliosides in the mechanisms underlying Alzheimer’s disease (AD), the most common form of dementia. Nutritional strategies targeting ganglioside sialylation—for example, through agglutinin-mediated modulation—have therefore attracted increasing research interest. In particular, wheat malt extract (WME), a food-derived source of wheat germ agglutinin (WGA) with high affinity for gangliosides, may influence molecular pathways involved in AD pathogenesis. Twelve-month-old female APPswe/PS1E9 transgenic mice, a model of AD, and wild-type (WT) littermates received WME or tap water for three weeks. Behavioral performance was subsequently assessed. Amyloid plaque burden and astrocyte activation were evaluated using Congo red staining and GFAP immunoreactivity, respectively. Gene expression of selected AD markers in the brain was quantified by RT–qPCR. Aged WT mice exhibited robust, region-specific molecular responses to WME, including upregulation of activity-dependent and synaptic plasticity genes (*Arc*, *Egr1*, *Bdnf*, *Syp*), enhancement of metabolic and insulin-related signaling (*Pgc1a*, *Sirt1*, *Igf1r*, *Irs2*), increased *Cldn5* expression, and reduced pro-inflammatory *Il1β* expression. APP/PS1 mice exhibited limited response to WME, suggesting more persistent transcriptional signatures of synaptic impairment, metabolic dysregulation, and neuroinflammation than in WT mice. We found no significant effects of WME treatment on amyloid plaque density and behavior in APP/PS1 mice. No effects on astrocyte activation were observed in either group. These findings demonstrate that dietary WME counteracts abnormal behaviors and molecular changes in neuron plasticity, metabolic, and vascular markers under conditions of normal aging but fails to improve the hallmarks of AD pathology. This highlights the potential of WGA-containing nutrients as a preventive nutritional approach targeting pathogenic mechanisms of aging and, potentially, AD pathology.

## 1. Introduction

Alzheimer’s disease (AD) is a progressive neurodegenerative disorder in which early synaptic failure progresses to widespread neuronal dysfunction, accompanied by cerebral hypometabolism, chronic neuroinflammation, and progressive disruption of blood–brain barrier (BBB) integrity [1,2]. One of the key molecular events implicated in the pathogenesis of AD is the amyloidogenic processing of amyloid precursor protein (APP), which occurs predominantly within lipid raft membrane domains enriched in cholesterol and gangliosides [3]. The major brain gangliosides—GM1, GD1a, GD1b, and GT1b—account for approximately 97% of total brain gangliosides and share a common core structure, differing in the number and position of sialic acid residues [4,5]. In AD, GM1 and GM2 accumulate in lipid rafts [6] and act as binding sites that promote amyloid-β aggregation [7]. Although overall ganglioside levels decline in AD [8], the relative proportion of GM1 is increased [9]. Consistently, inhibition of ganglioside synthesis reduces neuronal vulnerability to Aβ toxicity [10], supporting experimental evidence that gangliosides facilitate Aβ accumulation on neuronal membranes by modulating its spatial organization [11].

Genetic depletion of gangliosides similarly attenuates Aβ pathology, even in aggressive transgenic AD models [12]. In particular, st3gal5-deficient (ST3^−^/^−^) mice, which lack major brain gangliosides, crossed with 5XFAD mice expressing mutant human amyloid precursor protein and presenilin-1, exhibited markedly reduced amyloid plaque burden and neuroinflammation while preserving neuronal and synaptic integrity. These ST3^−^/^−^5XFAD mice also demonstrated significantly improved cognitive performance compared with 5XFAD controls, reaching levels comparable to non-transgenic wild-type (WT) mice. Comparable protective effects have been achieved pharmacologically; for example, *Limax flavus* agglutinin, which selectively binds α-sialylated gangliosides, disrupts Aβ–membrane interactions and reduces neurotoxicity [12].

These findings suggest that targeting ganglioside-associated glycans with sialic acid–binding agglutinins is a viable strategy to modulate early amyloidogenic events. Importantly, agglutinins are widely present in natural food sources, where they may exert biologically relevant effects through dietary exposure. In this context, wheat malt represents a promising candidate, as it naturally contains wheat germ agglutinin (WGA), a lectin with high affinity for sialic acid and N-acetylglucosamine residues on neuronal surface glycoconjugates [13].

Like most lectins, WGA is relatively resistant to degradation by digestive enzymes and gut microbiota, allowing a substantial proportion of orally ingested WGA to pass through the gastrointestinal tract intact [14]. Furthermore, morphological and tracer studies using WGA conjugates demonstrated that WGA can enter the brain from the bloodstream via adsorptive endocytosis at the luminal surface of BBB endothelial cells, exhibiting preferential transport in the blood-to-brain direction [15,16]. Additional experimental evidence indicates that WGA binds to neuronal membranes, undergoes axonal and transsynaptic transport, and interacts with glycosylated proteins within the central nervous system [15,17]. These properties are particularly relevant in light of accumulating evidence that alterations in glycosylation, changes in lectin-binding glycoproteins, and disturbances in carbohydrate-dependent signaling within the central nervous system contribute to Alzheimer’s disease pathogenesis [18,19,20].

To date, only a limited number of studies have examined WGA in AD-related contexts. In preclinical research, WGA has been used primarily as a targeting ligand in drug-delivery systems. For example, intravenously WGA-conjugated liposomes enhanced BBB transport of curcumin and nerve growth factor, resulting in reduced Aβ deposition and improved neuronal survival in the hippocampus of AD rats [21]. Similarly, WGA conjugation enhanced the cerebral uptake of anti-Aβ antibodies and produced greater Aβ reduction than unconjugated antibodies following intranasal administration in the 5XFAD mouse model [22]. Other studies have focused on WGA-reactive glycoproteins as potential diagnostic markers, reporting altered WGA binding in cerebrospinal fluid and disease-associated changes in transferrin and acetylcholinesterase glycosylation [18,23].

While these findings demonstrate that WGA-based conjugates can produce significant cognitive improvements in animal models of AD and highlight disruptions in carbohydrate-dependent interactions and glycosylation patterns, they primarily inform strategies for targeted drug delivery and biomarker development rather than direct therapeutic applications. Nevertheless, these studies support the biological plausibility of WGA-mediated modulation of central nervous system function and underscore the need to investigate nutritional strategies. In this context, wheat malt emerges as a promising candidate. Its high WGA content, together with other bioactive components, may modulate key pathogenic processes associated with AD, including amyloid-β–membrane interactions, glycosylation dynamics, metabolic dysregulation, and neuroinflammatory signaling.

To explore this potential, the present study is the first to evaluate the effects of WGA-enriched cold wheat malt extract (WME) on behavioral performance and histological AD-like changes in the brain as a nutritional intervention for AD [24,25,26]. Although the composition of WME may vary depending on the wheat source and place of origin [27,28], its general nutritional profile is well documented. Typical values per 100 g are as follows: water, 21.2 g; total carbohydrate, 71.9 g, of which available carbohydrate accounts for 70.5 g and total sugars for 44.4 g; protein (N × 6.25), 5.3 g; total dietary fiber, 1.4 g; total fat, 0.8 g, including saturated fatty acids (0.56 g), monounsaturated fatty acids (0.12 g), and polyunsaturated fatty acids (0.09 g); ash, 0.8 g; sodium, 11.3 mg. The energy value is 1329 kJ per 100 g [29,30]. In addition to its general nutritional profile, the concentration of WGA in the extract was specifically quantified to characterize the bioactive component of interest.

Following compositional characterization of the extract, its biological effects were investigated in vivo using APPswe/PS1E9 mice, a well-established model of AD [24,26]. In this study, 12-month-old mice were selected to represent a stage of advanced Alzheimer’s-like pathology [31]. This stage is characterized by pronounced amyloid deposition and astroglial activation. This approach allows us to assess whether a relatively short-term WME intervention can exert biological effects in conditions where pathological processes are already present [31]. We also analyzed the expression of genes involved in key processes related to Alzheimer’s disease (AD), whose expression has recently been shown to be altered in APPswe/PS1E9 mice. These included the pro-inflammatory marker interleukin-1 beta (*Il1β*) [24,26,32], genes regulating metabolic and insulin-dependent signaling, including peroxisome proliferator-activated receptor gamma coactivator-1α (*Pgc1a*), sirtuin 1 (*Sirt1*), insulin-like growth factor 1 receptor (*Igf1r*), and insulin receptor substrate 2 (*Irs2*) [25,26], which are closely associated with mitochondrial function and metabolic impairment in AD [33,34,35,36], claudin-5 (*Cldn5*) as a marker of BBB integrity [25,26,37] and plasticity-related genes including activity-regulated cytoskeleton-associated protein (*Arc*), early growth response protein 1 (*Egr1*), brain-derived neurotrophic factor (*Bdnf*), and synaptophysin (*Syp*), reflecting synaptic function and neuronal adaptability [25,38,39,40,41]. In addition, growth differentiation factor 15 (GDF15) was included as a marker of mitochondrial stress and metabolic adaptation, as it is induced under conditions of mitochondrial dysfunction, oxidative stress, and metabolic imbalance [42,43]. Increasing evidence suggests that GDF15 contributes to the regulation of metabolic and inflammatory pathways associated with aging and Alzheimer’s disease pathogenesis [44,45].

## 2. Results

### 2.1. WGA Characterization in Wheat Malt Extract

**Figure 1:**
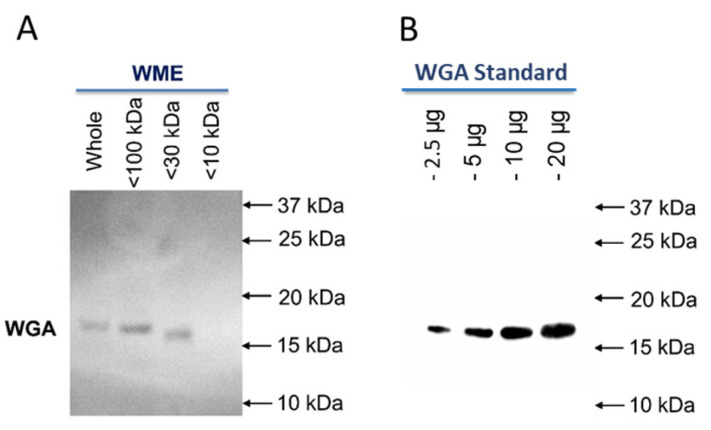
Western blot analysis of the presence of wheat germ agglutinin (WGA) in unfractionated vs. 0–100, 0–30, and 0–10 kDa fractions of wheat malt extracts (WME). WME were obtained and used as unmanipulated or after passage through 100, 30, and 10 kDa filters (**A**). Equal volumes were further analyzed for WGA as described in the Section 4. Antibody specificity was assessed using WGA standards at concentrations ranging from 2.5 μg to 20 μg (**B**).

It was reported that WGA has three isoforms, and it is present as 17 kDa or 18 kDa protein chains that usually form dimers in the solution [14]. We assessed the presence of WGA dimers and monomers in our WME using a fractionation strategy followed by Western blot analysis. Unfractionated WME or WME fractions that were passed through 100, 30, and 10 kDa filters were analyzed. WGA was detected in whole WME, <100 kDa, and <30 kDa WME fractions. WGA was not detected in the ≤10 kDa WME fraction (Figure 1A), as expected, since it has a larger molecular weight. Since the 17–18 kD dimer has a molecular weight exceeding 30 kDa (34–36 kDa), our analysis indicates the presence of monomers in our WME extracts as well. Quite interestingly, the >30 kDa band appeared to have better motility in polyacrylamide gel compared to that of the >100 kDa band (Figure 1). This might be related to its lower molecular weight of 17 kDa in monomer form compared to the 18 kDa in dimeric form. Importantly, we did not detect non-specific staining with the combination of our primary and secondary antibodies at 10–37 kDa (Figure 1; Figure S1) or 25–110 kDa. This finding makes our system suitable for routine assessment of WGA in WME extracts using the fast dot method, which revealed a WGA concentration of 0.9 ± 0.2 mg/mL. Thus, we identified a high concentration of WGA in the WME we used in our study.

### 2.2. Behavior Assessment

**Figure 2:**
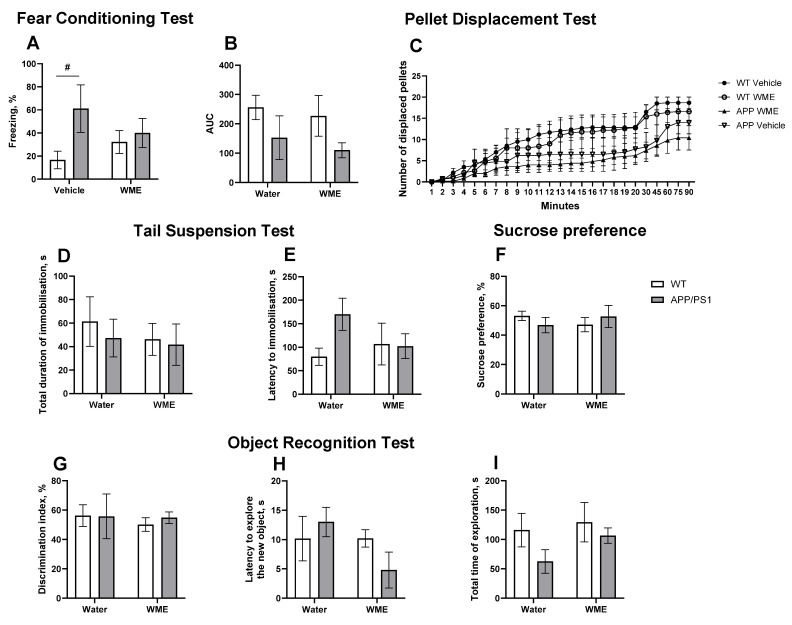
Behavioral performance of WT and APP/PS1 mice following chronic WME administration: fear conditioning performance (**A**); pellet displacement test: total activity expressed as AUC (**B**); temporal dynamics of pellet displacement across 90 min (**C**); tail suspension test (**D**,**E**); sucrose preference test (**F**); object recognition test (**G**–**I**). Data are presented as mean ± SEM (*n* = 5 mice per group). # *p* < 0.05 indicates exploratory, uncorrected Fisher’s LSD pairwise comparisons in the absence of significant main or interaction effects.

The results of the fear conditioning test are shown in Figure 2A. Two-way ANOVA revealed no significant main effects of genotype or treatment and no genotype × treatment interaction (F (1, 16) = 4.466, *p* = 0.0506; F (1, 16) = 0.05053, *p* = 0.8250, and F (1, 16) = 2.202, *p* = 0.1573, respectively). APP/PS1 transgenic mice in the control group exhibited significantly higher freezing levels than WT mice (*p* = 0.0228, uncorrected Fisher’s LSD test), suggesting increased anxiety in the mutants. Chronic administration of WME did not significantly affect the duration of freezing behavior in either WT or APP/PS1 mice. However, WME-treated APP/PS1 mice displayed lower freezing levels compared with untreated APP/PS1 mice, although this difference did not reach statistical significance (*p* = 0.6589, uncorrected Fisher’s LSD test). Notably, freezing responses were comparable between APP/PS1 and WT mice following WME exposure.

The results of the pellet displacement test are shown in Figure 2B,C. Post hoc multiple comparisons (Tukey’s test and uncorrected Fisher’s LSD) did not reveal statistically significant differences between individual groups at any time point (*p* > 0.9999). Two-way ANOVA of the time course of pellet displacement over 90 min revealed significant main effects of time (F (1, 16) = 11.27, *p* < 0.0001) and genotype (F (1, 16) = 30.59, *p* < 0.0001), while the time × genotype interaction was not significant (F (1, 16) = 0.3732, *p* > 0.9999). These findings indicate that although overall effects of time and genotype were detected across the dataset, they were not driven by discrete pairwise group differences.

In the tail suspension test, the total duration of immobilization (Figure 2D) and the latency to immobilization (Figure 2E) were measured. A two-way ANOVA revealed no statistically significant effects of treatment, genotype, or interaction on any parameters (F (1, 16) = 0.3205, *p* = 0.5792; F (1, 16) = 0.2576, *p* = 0.6187, and F (1, 16) = 0.06703, *p* = 0.7990 as the main effect for the total duration of immobilization and F (1, 16) = 2.267, *p* = 0.1516; F (1, 16) = 0.4263, *p* = 0.5231 and F (1, 16) = 1.843, *p* = 0.1934 as the main effect for the latency to immobilization). The sucrose preference test (Figure 2F) also revealed no significant effects of genotype, treatment, or interaction, according to a two-way ANOVA (F(1, 16) = 0.004729, *p* = 0.9460; F (1, 16) = 4.729, *p* = 0.9946, and F (1, 16) = 1.211, *p* = 0.2875). Similarly, the object recognition test measured discrimination index (%), time to novel object exploration, and total exploration time (Figure 2G–I). A two-way ANOVA revealed no significant effects of genotype, treatment, or their interaction on any of these parameters, indicating no statistically significant differences between groups (F (1, 16) = 0.06638, *p* = 0.8000; F (1, 16) = 0.1896, *p* = 0.6691, and F (1, 16) = 0.09953, *p* = 0.7565. F (1, 16) = 0.1743, *p* = 0.6819; F (1, 16) = 1.765, *p* = 0.2027 and F (1, 16) = 1.794, *p* = 0.1992. F (1, 16) = 2.069, *p* = 0.1696; F (1, 16) = 1.175, *p* = 0.2945 and F (1, 16) = 0.3349, *p* = 0.5708 respectively). Together, these results indicate that three-week WME consumption had no measurable effects on depressive-like behavior or hippocampus-dependent performance.

### 2.3. Effects of Treatment with WME on Plaque Deposition in the Brain

**Figure 3:**
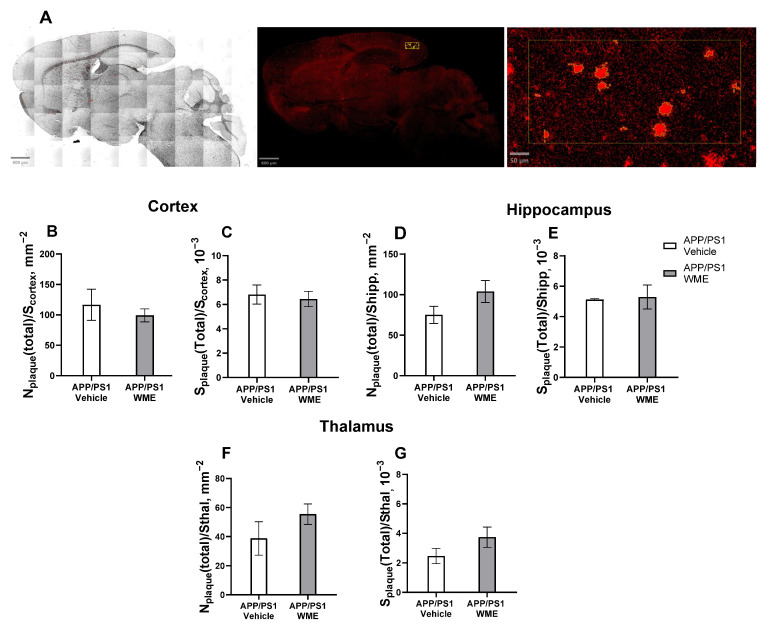
Representative Congo red-stained sagittal section of the brain from an APP/PS1 mouse (**A**): left—T-PMT signaling merged with Congo red image, amyloid deposits are shown in red, scale bar = 800 µm; middle and right—representative tile-scan images of Congo red-stained brain sections demonstrating amyloid plaques deposition in APP/PS1 mice, scale bar = 800 µm (middle) and 50 µm (right). Amyloid plaque density and total plaque-covered area in the cortex (**B**,**C**), hippocampus (**D**,**E**), and thalamus (**F**,**G**) of APP/PS1 mice treated with WME: (**B**,**D**,**F**) panels represent plaque density (N_plaques_/S_region_, mm^−2^), and (**C**,**E**,**G**) panels show total plaque-covered area (S_plaques_/Total S_region_, 10^−3^); data are presented as mean ± SEM (*n* = 5 mice per group).

Figure 3A and Figure S2A,B show representative Congo red–stained brain sections demonstrating extensive amyloid deposition in APP/PS1 mice. Quantitative analyses of amyloid plaque deposition in the cortex, hippocampus, and thalamus are presented in Figure 3B–G. Overall, the Mann–Whitney test revealed no significant difference in total plaque density across the cortex, hippocampus, and thalamus (*p* > 0.9999, *p* = 0.7857, and *p* = 0.2500, respectively). Specifically, the total plaque density, defined as the total number of amyloid plaques normalized to the analyzed brain region area (mm^2^), and the total plaque burden, defined as the total plaque-covered area normalized to the analyzed brain region area (×10^3^), showed no significant differences between groups in the cortex, hippocampus, or thalamus (*p* > 0.05, two-way ANOVA).

### 2.4. The Impact of the WME Treatment on the Number of GFAP-Immunoreactive Regions in the Brain

**Figure 4:**
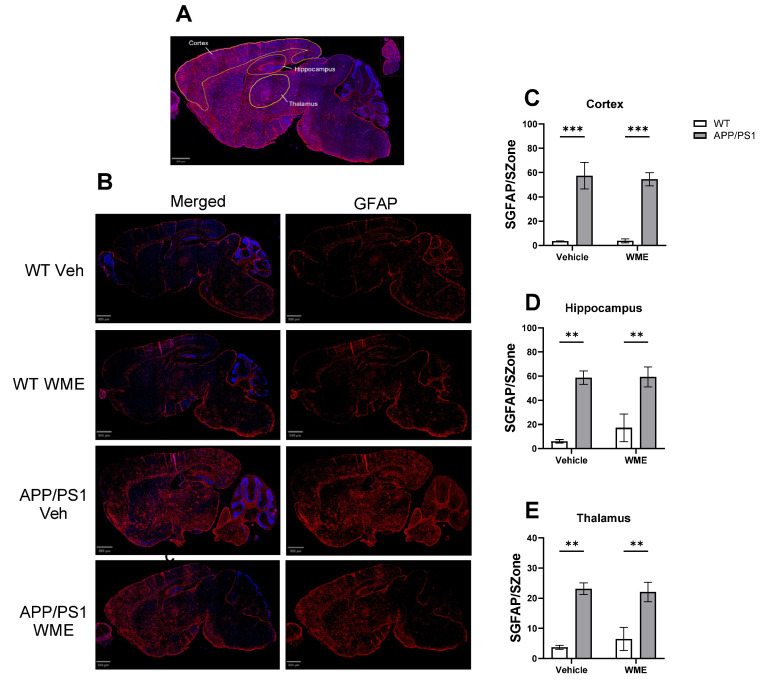
Immunohistochemical analysis of astrocyte activation in brain regions of WT and APP/PS1 mice. Representative images of mouse brain sections immunostained for GFAP (red), an astrocytic marker, and merged with DAPI nuclear staining (blue), showing representative regions of interest (ROIs) in the cortex, hippocampus, and thalamus, scale bar = 800 µm (**A**). Representative image of a whole-brain stained with anti-GFAP staining (red signal), as a marker of astrocytes, merged with DAPI nuclei staining (blue signal), the scale bar = 800 µm (**B**). Immunohistochemical analysis of astrocyte activation, GFAP immunoreactivity in cortex (**C**), hippocampus (**D**), and thalamus (**E**) of WT and APP/PS1 mice after WME treatment and the control group. Data are presented as mean ± SEM (*n* = 5 mice per group). ** *p* < 0.01, *** *p* < 0.001 indicate effects identified following ANOVA with post hoc testing.

Astroglial activation was evaluated in the prefrontal cortex, hippocampus, and thalamus to characterize the effects of chronic WME feeding on the area of GFAP-immunoreactive regions in APP/PS1 mice (Figure 4 and Figure S3). Representative whole-brain GFAP-immunostained images (Figure 4A,B) illustrate astrocyte distribution across experimental groups. Compared with WT mice, APP/PS1 animals exhibited a pronounced increase in GFAP immunoreactivity in the cerebral cortex, hippocampus, and thalamus, whereas WME treatment did not produce any evident changes in either genotype.

Consistent with these qualitative observations, quantitative analyses across all examined regions revealed a significant main effect of genotype, while neither treatment nor genotype-by-treatment interaction reached statistical significance (Figure 4C–E). In the prefrontal cortex, two-way ANOVA demonstrated a significant effect of genotype (F (1, 11) = 48.53, *p* = 0.0001), with no effect of treatment or interaction (F (1, 11) = 0.03180, *p* = 0.8617 and F (1, 11) = 0.04708, *p* = 0.8322, respectively). Fisher’s LSD post hoc analysis confirmed a significantly larger GFAP-positive area in APP/PS1 mice than in WT controls in both the placebo-treated (*p* = 0.0008) and WME-treated (*p* = 0.0002) groups (Figure 4C).

Similarly, in the hippocampus, a significant main effect of genotype was observed (F (1, 11) = 24.86, *p* = 0.0004, two-way ANOVA). In contrast, treatment and interaction effects were not significant (F (1, 11) = 0.3892, *p* = 0.5455 and F (1, 11) = 0.3026, *p* = 0.5932, respectively). Post hoc comparisons again demonstrated elevated GFAP immunoreactivity in APP/PS1 mice in both control (*p* = 0.0050) and WME-treated (*p* = 0.0040) groups (Figure 4D). In the thalamus, two-way ANOVA revealed a significant genotype effect (F (1, 11) = 26.24, *p* = 0.0003), with no significant treatment or interaction effects (F (1, 11) = 0.06090, *p* = 0.8096 and F = 0.3271, *p* = 0.5789, respectively). Fisher’s LSD test confirmed increased GFAP-positive area in APP/PS1 mice compared with WT animals in both vehicle-treated (*p* = 0.0042) and WME-treated (*p* = 0.0034) groups (Figure 4E).

Overall, these findings indicate that astrocytosis in APP/PS1 mice is strongly genotype-dependent and that chronic WME administration does not modulate astroglial activation in the prefrontal cortex, hippocampus, or thalamus.

### 2.5. Region-Specific Modulation of Inflammatory and Plasticity-Related Gene Expression by WME in the Cortex of APP/PS1 and WT Mice

The panel included markers of synaptic plasticity (*Arc*, *Egr1*, *Bdnf*), metabolic regulators (*Pgc1a*, *Sirt1*, *Igf1r*, *Irs2*), a synaptic vesicle protein (*Syp*), a pro-inflammatory cytokine (*Il1β*), and a BBB integrity marker (*Cldn5*).

**Figure 5:**
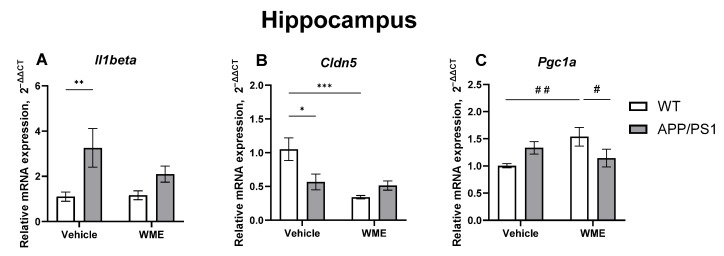
Relative mRNA expression of selected markers related to synaptic plasticity, metabolism, inflammation, and BBB integrity in the hippocampus of WT (white bars) and APP/PS1 (grey bars) mice: *Il1β* (**A**), *Cldn5* (**B**), *Pgc1a* (**C**). Data are shown as mean ± SEM, * *p* < 0.05, ** *p* < 0.01, *** *p* < 0.001 indicate effects identified following ANOVA with post hoc testing; # *p* < 0.05, ## *p* < 0.01 indicates exploratory, uncorrected Fisher’s LSD pairwise comparisons in the absence of significant main or interaction effects.

In the hippocampus, analysis of *Il1β* expression revealed a significant main effect of genotype (F (1, 16) = 14.02, *p* = 0.0018, two-way ANOVA). Uncorrected Fisher’s LSD post hoc test confirmed higher *Il1β* levels in APP/PS1 mice compared with WT animals in the vehicle group (*p* = 0.0021, Figure 5A). For *Cldn5*, two-way ANOVA identified significant main effects of treatment (F (1, 16) = 10.23, *p* = 0.0056) as well as a significant genotype x treatment interaction (F (1, 16) = 7.56, *p* = 0.0143). Post hoc analysis revealed higher *Cldn5* expression in WT mice compared with APP/PS1 mice under vehicle conditions (*p* = 0.0121). In WT animals, WME administration significantly reduced *Cldn5* expression compared with untreated control (*p* = 0.0004, Figure 5B). Notably, following WME exposure, *Il1β* and *Cldn5* expression levels were comparable between WT and APP/PS1 mice.

Expression of *Pgc1a* also demonstrated a significant genotype × treatment interaction (F (1, 16) = 7.69, *p* = 0.0136; two-way ANOVA), whereas neither main effect reached significance independently (F (1, 16) = 0.05931, *p* = 0.8107 and F (1, 16) = 1.749, *p* = 0.2046; two-way ANOVA). Fisher’s LSD post hoc analysis showed that WME treatment significantly increased *Pgc1a* expression in WT mice (*p* = 0.0075). In contrast, WME-treated APP/PS1 mice exhibited lower *Pgc1a* expression compared with WME-treated WT animals (*p* = 0.0467, Figure 5C).

**Figure 6:**
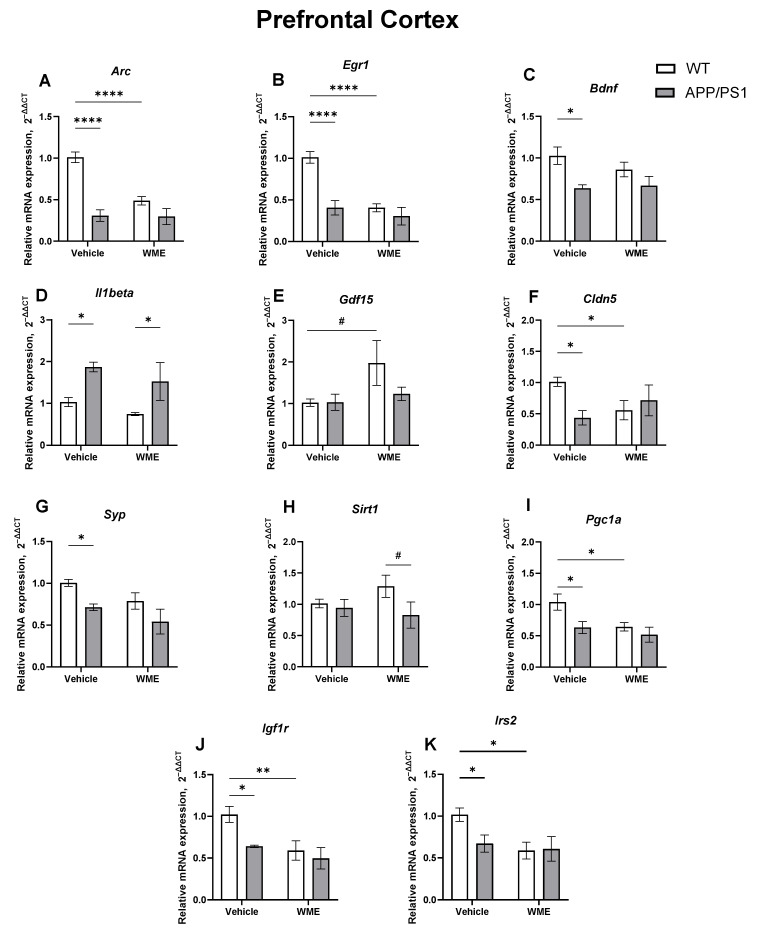
Relative mRNA expression of *Arc* (**A**), *Egr1* (**B**), *Bdnf* (**C**), *Il1β* (**D**), *Gdf15* (**E**), *Cldn5* (**F**), *Syp* (**G**), *Sirt1* (**H**), *Pgc1a* (**I**), *Igf1r* (**J**), and *Irs2* (**K**) markers associated with synaptic plasticity, metabolism, inflammation, and BBB integrity in the prefrontal cortex of WT (white bars) and APP/PS1 (grey bars) mice. Data are shown as mean ± SEM, * *p* < 0.05, ** *p* < 0.01, **** *p* < 0.000 indicate effects identified following ANOVA with post hoc testing; # *p* < 0.05 indicates exploratory, uncorrected Fisher’s LSD pairwise comparisons in the absence of significant main or interaction effects.

In the prefrontal cortex, two-way ANOVA of *Arc* expression revealed significant main effects of genotype (F (1, 16) = 37.23, *p* < 0.0001) and treatment (F (1, 16) = 13.32, *p* = 0.0022), as well as a significant genotype × treatment interaction (F (1, 16) = 12.29, *p* = 0.0029). Fisher’s LSD post hoc analysis demonstrated significant differences between WT and APP/PS1 mice in the vehicle-treated group (*p* < 0.0001), as well as between vehicle- and WME-treated WT mice (*p* < 0.0001, Figure 6A).

For *Egr1*, two-way ANOVA revealed significant main effects of genotype (F = 19.29, *p* = 0.0005) and treatment (F = 19.28, *p* = 0.0005), along with a significant genotype × treatment interaction (F = 9.91, *p* = 0.0062). Post hoc analysis confirmed higher *Egr1* expression in WT mice compared with APP/PS1 animals under control conditions (*p* < 0.0001), as well as a significant difference between control- and WME-fed WT mice (*p* < 0.0001, Figure 6B). Expression of *Bdnf* showed a significant main effect of genotype (F = 8.95, *p* = 0.0086), with Fisher’s LSD test revealing reduced *Bdnf* expression in APP/PS1 mice compared with WT animals in the control group (*p* = 0.0128, Figure 6C). A significant main effect of genotype was also observed for *Il1β* expression (F = 11.08, *p* = 0.0043). Post hoc comparisons demonstrated elevated *Il1β* levels in APP/PS1 mice relative to WT mice in both vehicle-treated (*p* = 0.0280) and WME-treated groups (*p* = 0.0360, Figure 6D).

For *Gdf15*, Fisher’s LSD analysis revealed increased mRNA expression in WME-treated WT mice compared with WT controls (*p* = 0.0330, Figure 6E). Analysis of *Cldn5* expression identified a significant genotype × treatment interaction (F = 5.26, *p* = 0.0357). Post hoc testing showed higher *Cldn5* expression in WT mice compared with APP/PS1 mice under control conditions (*p* = 0.0229), as well as increased expression in WME-treated WT mice compared with WT controls (*p* = 0.0498, Figure 6F). For *Syp*, two-way ANOVA revealed a significant main effect of genotype (F = 8.05, *p* = 0.0119). Post hoc analysis indicated reduced *Syp* expression in APP/PS1 mice compared with WT controls (*p* = 0.0469; Figure 6G). Although no significant main effects were detected for *Sirt1*, Fisher’s LSD test revealed increased *Sirt1* expression in WME-treated WT mice compared with APP/PS1 animals (*p* = 0.0485, Figure 6H).

Expression of *Pgc1a* showed significant main effects of treatment (F = 5.17, *p* = 0.0371) and genotype (F = 5.61, *p* = 0.0308). Post hoc analysis demonstrated higher *Pgc1a* expression in WT mice compared with both vehicle-treated APP/PS1 mice (*p* = 0.0219) and WME-treated WT mice (*p* = 0.0180, Figure 6I). Similarly, *Igf1r* expression exhibited significant main effects of treatment (F = 7.41, *p* = 0.0151) and genotype (F = 5.05, *p* = 0.0390). Fisher’s LSD testing revealed higher *Igf1r* expression in WT compared with APP/PS1 mice in the control group (*p* = 0.0226), as well as reduced expression in WME-treated WT mice relative to WT controls (*p* = 0.0077, Figure 6J).

Finally, *Irs2* expression demonstrated a significant main effect of treatment (F = 5.01, *p* = 0.0399), with post hoc analysis indicating higher expression in WT mice compared with APP/PS1 mice in the control group (*p* = 0.0433), and reduced expression in WME-treated WT mice compared with WT controls (*p* = 0.0103, Figure 6K).

## 3. Discussion

The present study examined the effects of wheat malt extract (WME), as a source of dietary wheat germ agglutinin (WGA), on behavioral performance in 12-month-old wild-type and APP/PS1 transgenic mice, as well as its influence on amyloid deposition, astrocyte activation, and brain transcriptional profiles. Our findings demonstrate that APP/PS1 mice exhibited widespread transcriptional alterations in both the hippocampus and PFC, with limited responsiveness to WME. In the hippocampus, changes included increased expression of *Il1β* and *Pgc1a*, together with reduced *Cldn5*. In the PFC, additional downregulation of *Arc*, *Erg1*, *Bdnf*, *Syp*, *Pgc1a*, *Igf1r*, and *Irs2* was observed. Furthermore, in APP/PS1 mice, three-week WME administration did not significantly alter behavioral performance, total amyloid plaque burden, or astrocyte activation.

Gene expression analysis in WT mice revealed distinct and biologically meaningful transcriptional modulation in response to WME. In both brain regions examined, WME influenced the expression of genes involved in metabolic regulation (*Pgc1a*, *Sirt1*) and blood–brain barrier integrity (*Cldn5*), in a genotype- and interaction-dependent manner. Notably, the prefrontal cortex exhibited a broader transcriptional response, encompassing activity-dependent immediate early genes (*Arc*, *Egr1*), inflammatory mediators (*Gdf15*), and components of insulin-related signaling (*Igf1r*, *Irs2*). The presence of multiple genotype × treatment interactions indicates that pre-existing, genotype-dependent molecular states shape the transcriptional response to WME, reflecting the differential sensitivity of brain tissue to dietary intervention.

Notably, many of the affected genes play key roles in processes increasingly recognized as critical in the early stages of Alzheimer’s disease pathogenesis. *Arc* and *Egr1* are immediate-early genes (IEGs) that are essential for activity-dependent synaptic plasticity [46]. At the same time, metabolic regulators, including *Pgc1a* and insulin signaling components, are closely linked to energy homeostasis and neuronal resilience [33].

Changes in *Cldn5* expression further suggest that WME can influence pathways associated with BBB regulation [47], even in the absence of overt structural abnormalities. WME induced region-specific changes in Cldn5 expression, with opposite trends observed in the hippocampus and prefrontal cortex, suggesting differential regulation of BBB-related pathways across brain regions. Given the preserved astroglial status and behavioral performance, the WME-associated reduction in *Cldn5* expression in WT mice may reflect a modulation of BBB-related pathways without evident compromise of barrier integrity. To clarify the functional significance of the observed *Cldn5* modulation, future studies that directly assess BBB permeability, such as tracer assays or analysis of additional tight junction components [15,16], will be important to determine whether these molecular changes translate into functional alterations.

Concurrently, wild-type mice showed increased hippocampal *PGC-1α* expression in response to WME. *PGC-1α* is a key regulator of mitochondrial biogenesis and cellular energy metabolism [48,49], and its activation suggests a favorable metabolic shift supporting neuronal resilience. Reduced *PGC-1α* activity has been linked to neuronal vulnerability, energetic insufficiency, and impaired neurovascular coupling [33,50]. Thus, elevated hippocampal *Pgc1a* expression in wild-type animals treated with wheat malt extract may reflect enhanced metabolic capacity and energy support, consistent with the concept of early preventive intervention.

In the prefrontal cortex, WME administration induced a coordinated transcriptional response in WT mice, primarily affecting genes involved in neuronal activity, metabolic regulation, and blood–brain barrier-related pathways. The observed decrease in the expression of the immediate early genes Arc and Egr1 suggests modulation of activity-dependent transcriptional processes [38,51]. However, in the absence of direct functional readouts of synaptic or network activity, these changes cannot be interpreted as evidence of improved or impaired neuronal function [52]. Given that increased expression of these genes is often associated with heightened neuronal load or stress-related activation [53,54,55], their reduction in the absence of behavioral impairment is consistent with normalization of cortical activity.

WME treatment increased Gdf15 expression, a gene associated with cellular adaptation to stress and metabolic regulation [56,57]. This change may indicate the activation of adaptive defense mechanisms that support cellular homeostasis. At the same time, decreased *Cldn5* expression indicates modulation of signaling pathways associated with the BBB. Importantly, this effect was observed without concomitant astrocyte activation or functional impairment, suggesting regulatory adaptation rather than barrier disruption.

WME also reduced the expression of genes associated with metabolic processes and insulin signaling (*PGC-1α*, *Igf1r*, *Irs2*), suggesting a shift in metabolic signaling in the prefrontal cortex [58]. Given preserved behavioral performance and the absence of overt neuropathology, these changes are more consistent with fine-tuning of energy- and growth factor-related pathways than with metabolic failure. Notably, *Sirt1* expression remained unchanged, supporting the notion that WME does not induce a generalized stress response [59].

In parallel with molecular modulation, behavioral analysis revealed no statistically significant treatment effects. This apparent mismatch between transcriptional changes and the lack of detectable behavioral or structural effects may be explained by the fact that gene expression changes can occur early and do not always lead to immediate functional outcomes, especially within a short intervention period. In APP/PS1 mice, the advanced stage of pathology may further limit the ability of these molecular changes to affect already established features. In contrast, in WT mice, preserved plasticity may allow such transcriptional responses to occur without immediate functional consequences. However, several measures exhibited consistent directional trends. In particular, WME-treated APP/PS1 mice showed a tendency toward reduced freezing behavior in the fear conditioning test, altered stress-coping responses in the pellet displacement test, and modest shifts in exploration and repetitive behaviors in the object recognition test. Although these trends did not reach statistical significance, their directionality aligns with the observed transcriptional changes and suggests that molecular modulation may precede detectable behavioral outcomes.

Interestingly, the untreated APP/PS1 group showed prolonged freezing behavior during the fear conditioning test. The observation of increased freezing in untreated APP/PS1 mice may appear formally counterintuitive; however, enhanced freezing during contextual fear conditioning does not necessarily indicate superior hippocampus-dependent memory. Freezing behavior reflects not only contextual learning, but also anxiety, emotional reactivity, stress responsiveness, and amygdala-dependent fear processing [60,61]. Several non-mutually exclusive mechanisms may therefore explain the elevated freezing observed in APP/PS1 mice. First, APP/PS1 mice display increased anxiety-like behavior and altered emotionality at 12 months of age [24,25,26,62,63]. Increased freezing may thus reflect heightened threat sensitivity and passive coping behavior rather than improved learning [64]. Second, altered stress responsiveness may contribute to these changes, as APP/PS1 mice exhibit elevated corticosterone levels from 9 months onward [65], while glucocorticoid dysregulation is known to exacerbate Alzheimer-like pathology and increase freezing behavior [60,61,66]. The tendency of WME treatment to reduce freezing, together with its neuroprotective effects, further supports a role for stress responsivity in these behavioral differences. Finally, abnormalities in amygdala-dependent fear circuitry reported in APP/PS1 mice [67,68] may exaggerate fear expression independently of memory performance [69,70] and impair contextual discrimination, promoting fear generalization and elevated freezing [71]. Importantly, our studies and previously published reports consistently demonstrated hippocampus-dependent cognitive deficits in 12-month-old APP/PS1 mice [24,25,26,62,63,65], making it unlikely that the increased freezing observed here reflects improved memory function.

Consistent with the amyloidogenic nature of the APP/PS1 model [72], amyloid plaque accumulation was evident across all brain regions examined. However, the overall amyloid plaque density and plaque-covered area remained unchanged after WME treatment. It should be noted that Congo red staining primarily detects mature, fibrillar amyloid deposits and may underestimate early or diffuse plaques, as reported in studies by Strekalova and colleagues and others. Therefore, subtle changes in less compact amyloid forms cannot be excluded [26]. Similarly, GFAP-based immunohistochemistry revealed marked astrocytosis in APP/PS1 mice compared to wild-type controls in the brain areas investigated, which was not altered by WME administration. The absence of detectable effects on amyloid burden and astrogliosis may be related to the relatively short treatment duration. Nevertheless, the observed molecular changes suggest that longer-term or earlier WME administration may have greater potential to influence the progression of AD-like pathology.

These findings, however, should be interpreted in light of several important limitations of the present study. First, 12-month-old APP/PS1 mice represent an advanced stage of pathology, potentially reducing responsiveness to short-term interventions. Second, the relatively small sample size (*n* = 5 per group) may limit the ability to detect subtle behavioral or pathological effects. Third, only female mice were included, preventing assessment of sex-specific responses relevant to Alzheimer’s disease. In addition, WME was administered ad libitum, and individual intake was not quantified, limiting precise estimation of exposure levels. These factors likely contributed to the modest pathological and functional effects observed. Furthermore, the use of uncorrected Fisher’s LSD testing may have increased the risk of Type I errors. Therefore, results showing nominal statistical significance in the absence of significant main or interaction effects should be interpreted as exploratory and hypothesis-generating rather than confirmatory.

Taken together, the presented data support a mechanistic framework in which three-week WME consumption does not reverse established Alzheimer’s disease pathology, but instead selectively modulates molecular pathways that may precede irreversible neurodegeneration. The observed dissociation between transcriptional changes and the lack of effects on amyloid burden, astrocytosis, and overt behavior underscores the importance of targeting early disease mechanisms rather than late-stage pathology. These findings highlight the potential of nutritionally relevant interventions to influence molecular processes at stages when neurodegeneration may still be preventable.

It should also be considered that WME itself represents a complex mixture of bioactive components, and the observed effects, therefore, cannot be attributed exclusively to WGA. Although WGA is a key lectin with known glycan-binding properties, other constituents of the extract may also contribute to the observed molecular responses. Consequently, the effects described here likely reflect the combined activity of several compounds within WME rather than the action of a single molecule. Given this complexity, although no adverse effects were observed in the present short-term study, further investigations, including longer-term interventions and direct functional assessments, are required to evaluate the safety profile and biological impact of WME comprehensively.

## 4. Materials and Methods

### 4.1. Animals and Study Flow

Twelve-month-old female APPswe/PS1E9 (APP/PS1; MMRRC Strain #034829-JAX, Jackson Laboratory, Bar Harbor, ME, USA) mice and their wild-type (WT) littermates on a C57BL/6 background were bred at the Laboratory of Genetic Technology and Gene Editing for Biomedicine and Veterinary in Belgorod, Russia. The animals were housed in groups of 3–5 per cage under standard laboratory conditions, including a reversed 12 h light/dark cycle (lights on at 21:00), ambient temperature of 22 ± 1 °C, and humidity of 55%, with ad libitum access to food and water, or WME (see below). All experiments were performed according to the ethical guidelines of the U.S. Department of Health and Human Services (HHS) and the Registration of an Institutional Review Board (IRB) and were approved by the Ethics Committee of the Center for Life Sciences of Nazarbayev University (protocol No. 05-2023, 21 November 2023).

**Figure 7:**
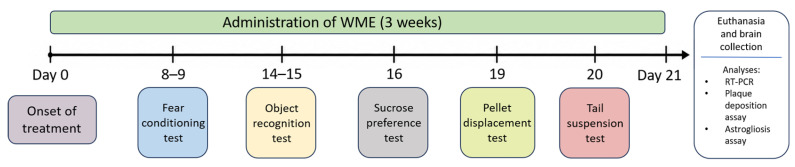
Schematic representation of experimental design. Mice received WME for 3 weeks starting from day 0. Behavioral testing was conducted during the treatment period at indicated time points: fear conditioning (days 8–9), object recognition (days 14–15), sucrose preference (day 16), pellet displacement (day 19), and tail suspension test (day 20). On day 21, animals were sacrificed, and brain tissue was collected for molecular and histological analyses.

The experimental design and timeline are schematically presented in Figure 7. Each experimental group included 5 mice; randomization was conducted by body weight before the onset of treatment. Beginning from day 0 (onset of treatment), groups of APP/PS1 and WT mice received WME instead of drinking water for three weeks. Based on a typical daily fluid intake of approximately 5 mL/day in adult mice and a measured WGA concentration of 0.9 ± 0.2 mg/mL in WME (see below), the estimated WGA intake was approximately 4.5 mg/day, corresponding to ~180 mg/kg/day for a 25 g mouse; this value should be considered an approximate estimate, as individual intake was not directly monitored. Control APP/PS1 and WT mice received drinking water.

Behavioral testing was conducted during the administration period on the following days with respect to the start of the treatment: fear conditioning (days 8–9), object recognition (days 14–15), sucrose preference (day 16), pellet displacement test (day 19), and tail suspension (day 20). On day 21, mice were sacrificed (see below), and brains were collected for RT-PCR and histological assessment of amyloid plaque formation (see below).

A sensitivity power analysis for the 2 × 2 between-subjects ANOVA design used in the study was performed a priori. With four groups and *n* = 5 mice per group, the design had 80% power to detect effects of approximately Cohen’s f = 0.67, corresponding to partial η^2^ ≈ 0.31, indicating that group size was sufficient to detect large effects expected based on previously published data [24,25,26].

### 4.2. Wheat Malt Extract Preparation and WGA Measurement

Factory-ground wheat malt (Soufflet Group, Nogent-sur-Seine, France) was mixed with the tap water used for animals’ housing for 3–4 min at a ratio of 1/5. For cold extraction, the mixture was incubated overnight at room temperature under constant stirring (150 rpm). After extraction, the suspension was centrifuged for 20 min at 4000 rpm to sediment insoluble material. The supernatant was then aseptically collected into sterile 50 mL centrifuge tubes and stored at +4 °C until use.

WGA concentration in the extract was measured by the dot method using nitrocellulose membrane (Thermo Scientific, cat#PI88025, Waltham, MA, USA) and non-conjugated rabbit anti-WGA antibodies (Abcam, Cambridge, UK, cat# ab178444, 1:1000) combined with goat HRP-conjugated anti-rabbit antibodies (Thermo Scientific, cat# 31460, 1:1000) and commercially available purified WGA protein as a standard (MP Biomedicals, Irvine, CA, USA, cat# 790162). To ensure the specificity of our assay for measuring WGA concentration by the dot method, the malt extract was passed through a 0.22 µm membrane filter and fractionated using 10, 30, and 100 kDa filters (Microcon, Merk, Darmstadt, Germany), as we did earlier [73].

The presence of WGA in WME fractions of 100, 30, and 10 kDa was assessed by Western blot analysis using the same primary and secondary antibodies as in the dot blot method described above, as in our previous studies for other proteins [74]. Briefly, the samples were treated with DTT to break S-S bonds and dimeric structures, and 30 µL of each sample was loaded onto a polyacrylamide gel. Anti-WGA antibodies were used at a dilution of 1:1000 in PBS with 5% BSA and incubated overnight at 4 °C. Secondary antibodies were used at a dilution of 1:1000 and incubated for 1 h at 4 °C. Antibody specificity was confirmed using WGA standard (MP Biomedicals; Cat. No. 08790162). Different concentrations of the standard, ranging from 2.5 μg to 20 μg, were tested by Western blotting under the same conditions as described above.

### 4.3. Behavioral Assays

#### 4.3.1. Fear Conditioning Test

The assessment of conditioned fear learning was carried out as described elsewhere [75,76,77]. The apparatus (Technosmart, Rome, Italy) consisted of a transparent plastic cubicle (25 cm × 25 cm × 50 cm) with a stainless-steel grid floor (2 mm rods spaced 0.9 cm apart). Following a 2.5-min acclimatization period, a single footshock was delivered through the grid (AC, 50 Hz; 0.7 mA; 1 s; Evolocus LLC, Tarrytown, NY, USA). Mice remained in the chamber for an additional 30 s after shock delivery and were then returned to their home cage. Memory recall was tested 24 h later. Freezing behavior was scored by visual observation during a 3 min recall session. The occurrence of freezing was assessed every 10 s; each observation was scored as freezing or not freezing, and the percentage of time spent freezing was calculated for all groups of mice [78].

#### 4.3.2. Object Recognition Test

The experimental procedure was conducted within a cubic apparatus (45 cm × 45 cm × 45 cm) under dim light of 5 lux, as described elsewhere [24,25,26]. During the initial day of the test, two identical objects (“brush,” approximately 7 cm × 4 cm × 3 cm) were positioned at diagonally opposite corners of the apparatus, and the rodents were allowed to explore freely for 15 min [78]. The following day, one of the stimuli was replaced with a novel item (“flower,” approximately 7 cm × 4 cm × 3 cm). The duration of object exploration was defined as the time when the mouse’s nose was oriented towards the object while positioned at a distance of less than 2 cm from it. The latency to explore the novel object and the duration of exploration of the two objects were recorded, and the discrimination index was computed using the following formula as described elsewhere [79]:

$$Object\;discrimination\;index=\;\frac{Time\;of\;exploration\;of\;the\;novel\;object}{Total\;exploration\;time}\times 100\%$$

#### 4.3.3. Sucrose Preference Test

Mice were given eight hours of free choice between two bottles of 1% sucrose solution and standard drinking water, as described elsewhere [80]. At the beginning and end of the period, the bottles were weighed, and consumption was calculated. The test began with the onset of the dark (active) phase of the animals’ cycle (i.e., at 9:00). To prevent possible effects of side preference on drinking behavior, the bottles’ positions in the cage were switched at 4 h (halfway through testing). No previous food or water deprivation was applied before the test. To minimize liquid spillage during the sucrose test, bottles were filled in advance and kept upside down for at least 12 h before testing. To balance the air temperature between the room and the drinking bottles, they were kept in the same room where testing took place. This measure prevents the physical effects of liquid leakage caused by rising air temperature and pressure inside the bottles when they are filled with liquids that are cooler than the room air. The percentage preference for sucrose was calculated using the following formula [79]:

$$Sucrose\;preference=\frac{{Volume}_{\left(Sucrose\;solution\right)}}{{Volume}_{\left(Sucrose\;solution\right)}+{Volume}_{\left(Water\right)}}\times 100\%$$

#### 4.3.4. Pellet Displacement Test

The tendency of rodents to remove small objects, such as stones or food pellets, from a tube placed inside the home cage is species-specific and depends on intact hippocampal function. In this test, a paper tube (4 cm internal diameter, 10 cm length) was filled with 20 food pellets and positioned in the center of the cage. Pellet displacement was scored every minute for the first 20 min, and then every 15 min thereafter, beginning at minute 30, following the procedure described elsewhere [26,81]. The test lasted 90 min in total.

#### 4.3.5. Tail Suspension Test

Mice were subjected to the tail suspension test by being hung by their tails with adhesive tape to a rod 50 cm above the floor for 6 min. Animals were tested in a dark room under red light. The latency to the first immobility episode, total duration of immobility, and number of immobility episodes were measured as described elsewhere [82]. In accordance with the commonly accepted criteria for immobility, immobility behavior was defined as the absence of any movement of the animals’ heads or bodies.

### 4.4. Euthanasia

Mice were terminally anesthetized by isoflurane using equipment from RWD Life Science Co. (Shenzhen, China). After confirmation of deep anesthesia, animals were perfused transcardially with ice-cold saline through the left ventricle. One half of the brain was removed, and the hippocampus and prefrontal cortex were dissected from one hemisphere and stored at −80 °C until further analysis, as previously described [83]. The remaining half of the brain was excised, fixed in formaldehyde overnight, and then fixed in formalin for 24 h before being processed for histological evaluation.

### 4.5. Brain Sectioning for Histological Assays

Brain tissue was washed three times for one hour and then dehydrated using graded ethanol solutions (75% for 1 h, 96% (I) for 5 min, 96% (II) for 5 min, 96% (III) for 5 min, 100% (I) for 5 min, and 100% (II) for 10 min). Each sample was incubated consecutively with 100% ethanol–chloroform (1:1) for 30 min, chloroform (I) for one hour, and chloroform (II) overnight, and embedded in paraffin (three times, for one hour each) at 60 °C, using a Leica EG1160 tissue embedding station (Leica Biosystems Inc., Nussloch, Germany). Paraffin sections were cut at 8 µm and mounted on polylysine-coated slides using a Leica RM 2265 microtome (Leica Biosystems Inc., Deer Park, IL, USA), as described previously [83].

### 4.6. Congo Red Staining, Plaque Microscopy, and Scoring of Amyloid Plaque Parameters

For tissue staining, the sections were deparaffinized in a xylene bath for 20 min, rehydrated with graded ethanol solutions (100% for 20 min, 95% for 5 min, and 50% for 5 min), and subsequently washed three times with deionized water for 5 min each. The sections were stained with 0.5% Congo red in 50% ethanol for 5 min and differentiated with 0.2% KOH in 80% ethanol for 3 min. Thereafter, the slices were embedded in the Epredia™ Immu-Mount™ water-based mounting medium (Thermo Fisher Scientific Inc., Kalamazoo, MI, USA). A total of 10 slices per animal were analyzed via confocal laser scanning microscopy (LSM880 in tile scan mode; Carl Zeiss, Oberkochen, Germany). To facilitate structural visualization, Congo red staining was combined with transmitted-light photomultiplier (T-PMT) imaging [84]. The processing of the slices for morphological analysis of β-amyloid deposits was conducted utilizing the QuPath 0.4.3-pixel classifier (Belfast, Northern Ireland, UK), an open-source software specifically designed for digital pathology image analysis. The images were meticulously double-checked to eliminate any artifacts and folds [85]. The analysis of the fluorescent images was performed in a single red channel, compared with the T-PMT-negative dots. The selection of regions with brightness exceeding the threshold was executed pixel by pixel. The total number of detected plaques was quantified, and the area of each plaque within each ROI was computed using the algorithm. The dimensions of these regions were determined and corroborated using a histology atlas [86]. The image analysis indicated the presence of amyloid plaques of various sizes. The density of each plaque type was calculated per mm^2^ in each of the investigated brain regions. Furthermore, the total area and number of plaques were documented.

### 4.7. Immunohistochemical Analysis of Astrocyte Activation

The immunostaining procedure was performed overnight in a humidified chamber at +4 °C, according to previously established protocols [87,88]. In summary, the brain sections, which were 8 µm in thickness, were subjected to staining with primary anti-GFAP (Glial fibrillary acidic protein) antibody (ab7260, Recombinant Full-Length Protein corresponding to Human/Mouse GFAP, Rabbit polyclonal (Abcam, diluted 1:1000) and secondary Goat anti-Rabbit IgG (H + L) antibodies (A11011, Alexa Fluor™ 568, Invitogen™, Thermo Fisher Scientific Inc., Carlsbad, CA, USA, diluted 1:1000). The nuclei were counterstained with DAPI (62248, Thermo Fisher Scientific Inc., Carlsbad, CA, USA, diluted 1:1000) and subsequently embedded in the Epredia™ Immu-Mount™ water-based mounting medium (Thermo Fisher Scientific Inc., Kalamazoo, MI, USA).

### 4.8. RNA Extraction, cDNA Synthesis, Primer Design, and Real-Time Polymerase Chain Reaction

Total RNA from prefrontal cortex and hippocampal tissue samples was isolated using QIAzol^®^ lysis reagent (QIAGEN Sciences Inc., Germantown, MD, USA). Tissue was placed in 1 mL of QIAzol, homogenized using a TissueRuptor homogenizer (QIAGEN Sciences Inc., Germantown, MD, USA) for two 30 s cycles at half speed, and then placed on ice for one minute after each homogenization. Homogenized samples were centrifuged for 15 min at 12,000× *g* and 4 °C to remove residual cellular debris. Chloroform was added to the homogenized sample, which was then centrifuged for 15 min at 12,000× *g* and 4 °C. The aqueous phase was carefully discarded. RNA precipitation was performed by adding ethanol, and the resulting RNA pellet was washed and purified using the RNeasy Mini Kit (QIAGEN Sciences Inc., Germantown, MD, USA). RNA concentration was measured using a NanoDrop spectrophotometer (Thermo Fisher Scientific, Waltham, MA, USA).

Total RNA (1 μg) was converted to cDNA. First-strand cDNA synthesis was performed using random primers and the QuantiTect Reverse Transcription Kit (QIAGEN Sciences Inc., Germantown, MD, USA). The reaction was carried out in an Eppendorf Mastercycler^®^ Gradient thermal cycler (Eppendorf, Hamburg, Germany) using the following temperature program: 68 °C for 5 min, 42 °C for 60 min, and 70 °C for 10 min.

The expression levels of target genes were identified using predesigned gene-specific primers. The housekeeping glyceraldehyde 3-phosphate dehydrogenase (*Gapdh*) gene was used as a reference. The sequences of designed primers are shown in Table 1.

The expression levels of immune activation marker *Il1β* [32], metabolic and insulin-dependent signaling markers (*Pgc1a*, *Sirt1*, *Igf1r*, *Irs2*) [33,34,35,36], BBB integrity marker (*Cldn5*) [37], and cellular plasticity markers (*Arc*, *Egr1*, *Bdnf*, *Syp*) [38,39,40,41] were determined using real-time polymerase chain reaction (RT-PCR). RT-PCR was performed on 7900 HT Fast (Applied Biosystems, Foster City, CA, USA) instrument using 384-well plate in a 10 µL reaction volume containing 5 L of 2 × SYBR Green Universal Master Mix (Applied Biosystems, Foster City, CA, USA), 3 µL RNase-free water, specific primers pair at a concentration of 20 pmol/µL (1 µL), and cDNA (1 µL). The initial denaturation step for RT-PCR was performed at 95 °C for 5 min, followed by 40 cycles of denaturation at 95 °C for 20 s, annealing at 60 °C for 30 s, and elongation at 68 °C for 30 s. The expression data were analyzed using SDS 2.4 software (Applied Biosystems, Foster City, CA, USA).

### 4.9. Statistical Analysis

The data were analyzed using GraphPad Prism 10.4.0 (San Diego, CA, USA). For behavior assessment, a two-way ANOVA was performed, and Tukey’s and Fisher’s LSD post hoc tests were used to compare means between groups. Plaque density in the brain regions (cortex, hippocampus, and thalamus) was analyzed using two-way ANOVA followed by Šídák’s multiple comparisons test. Total plaque density and total plaque-covered area in the above-mentioned areas of the brain were analyzed using the non-parametric Mann–Whitney test. To evaluate the number of GFAP-positive cells in the brain and the expression levels of molecular markers of neuroplasticity and inflammation, a two-way ANOVA was performed, followed by Fisher’s LSD post hoc test. The normality of data distribution and homogeneity of variances were assessed prior to analysis using the Shapiro–Wilk test. The selection of post hoc tests reflected a balance between controlling Type I error (Tukey’s and Šídák’s tests) and maintaining sensitivity to detect biologically relevant differences in exploratory analyses (Fisher’s LSD). Sample sizes (*n*) are indicated in the figure legends. For ANOVA-based analyses, F values are reported together with the corresponding degrees of freedom and *p* values. The significance level was set at 95% (*p* < 0.05). Data are presented as the mean ± SEM; group sizes are indicated in the Section 4.1.

## 5. Conclusions

In conclusion, this study demonstrates that wheat malt extract, as a nutritionally relevant plant-derived intervention, exerts robust molecular effects in the context of aging. Although short-term dietary WME intake in aged APP/PS1 mice with established pathology did not alter cognitive performance, amyloid plaque burden, or astroglial activation, it induced pronounced transcriptional modulation of pathways involved in synaptic plasticity, metabolic regulation, neuroinflammation, and blood–brain barrier function in aged WT mice, suggesting that the biological responsiveness to WME is influenced by disease stage and preserved physiological plasticity. These findings highlight WME’s capacity to modulate molecular pathways implicated in Alzheimer’s disease and support its potential as a dietary strategy targeting early pathogenic mechanisms. Together with other emerging approaches [25,26], this provides a promising framework for developing effective early interventions in AD and identifies new avenues for preventive strategies. Further studies employing long-term dietary interventions and preclinical models, particularly at earlier disease stages, are warranted to fully elucidate their preventive efficacy and translational potential.

## Figures and Tables

**Table 1:** The sequences of the designed primers.

№	Gene	Primer Sequence 5′–3′	
1	*Gapdh*	For	ATGACCACAGTCCATGCCATC
		Rev	GAGCTTCCCGTTCAGCTCTG
2	*Il-1β*	For	TTGAAGTTGACGGACCCCAA
		Rev	ATGTGCTGCTGCGAGATTTG
3	*Tubb3*	For	CGAGACCTACTGCATCGACA
		Rev	CATTGAGCTGACCAGGGAAT
4	*Gdf15*	For	GACTGTGCAGGCAACTCTTG
		Rev	CGATACAGGTGGGGACACTC
5	*Sirt1*	For	TTGCAACAGCATCTTGCCTG
		Rev	CCTAGGGCACCGAGGAACTA
6	*Cldn5*	For	GAGTTCAGCTTCCCGGTCAA
		Rev	CTCCCGCCCTTAGACATAGTTC
7	*Arc*	For	CTGAGCCACCTAGAGGAGTACT
		Rev	AACTCCACCCAGTTCTTCACGG
8	*Bdnf*	For	CGGCGCCCATGAAAGAAGTA
		Rev	AGACCTCTCGAACCTGCCCT
9	*Egr1*	For	AGCAGCACCTTCAACCCTCAGG
		Rev	GAGTGGTTTGGCTGGGGTAACT
10	*Syp*	For	TGCCAACAAGACGGAGAGTG
		Rev	TAGTGCCCCCTTTAACGCAG
11	*Pgc1a*	For	CCAAAGGATGCGCTCTCGTTCA
		Rev	CGGTGTCTGTAGTGGCTTGACT
12	*Igf1r*	For	GTGGGGGCTCGTGTTTCTC
		Rev	GATCACCGTGCAGTTTTCCA
13	*Irs2*	For	CTGCGTCCTCTCCCAAAGTG
		Rev	GGGGTCATGGGCATGTAGC

## Data Availability

Data are available on reasonable request. To access the data, contact Tatyana Strekalova (tatslova@gmail.com) and Sholpan Askarova (shaskarova@nu.edu.kz).

## Supplementary Materials

The following supporting information can be downloaded at https://www.mdpi.com/article/10.3390/ijms27114994/s1.

## Abbreviations

The following abbreviations are used in this manuscript:

AD	Alzheimer’s disease
APP/PS1	APPswe/PS1E9 mice
WT	Wild-type
5XFAD	5XFAD transgenic mice
Aβ	Amyloid-beta peptide
APP	Amyloid precursor protein
WME	Wheat malt extract
WGA	Wheat germ agglutinin
BBB	Blood-brain barrier
PBS	Phosphate-buffered saline
PFC	Prefrontal cortex
GFAP	Glial fibrillary acidic protein
ROI	Region of interest
Gapdh	glyceraldehyde 3-phosphate dehydrogenase
Il1β	Interleukin 1 beta
Il6	Interleukin 6
Gdf15	Growth Differentiation Factor 15
Sirt1	Sirtuin 1
Cldn5	Claudin 5
Bdnf	Brain-Derived Neurotrophic Factor
Pgc1a	Peroxisome proliferator-activated receptor gamma coactivator 1-alpha
Arc	Activity-regulated cytoskeleton-associated protein
Egr1	Early Growth Response 1
Igf1r	Insulin-like growth factor-1 receptor
Irs2	Insulin receptor substrate 2
