# Supplementary figures and images for: Effects of Wheat Malt Extract on Molecular and Behavioral Markers in Aged APP/PS1 and Wild-Type Mice
Source: Int J Mol Sci. 2026 May 30;27(11):4994. doi: 10.3390/ijms27114994 (PMC13256562; doi:10.3390/ijms27114994)

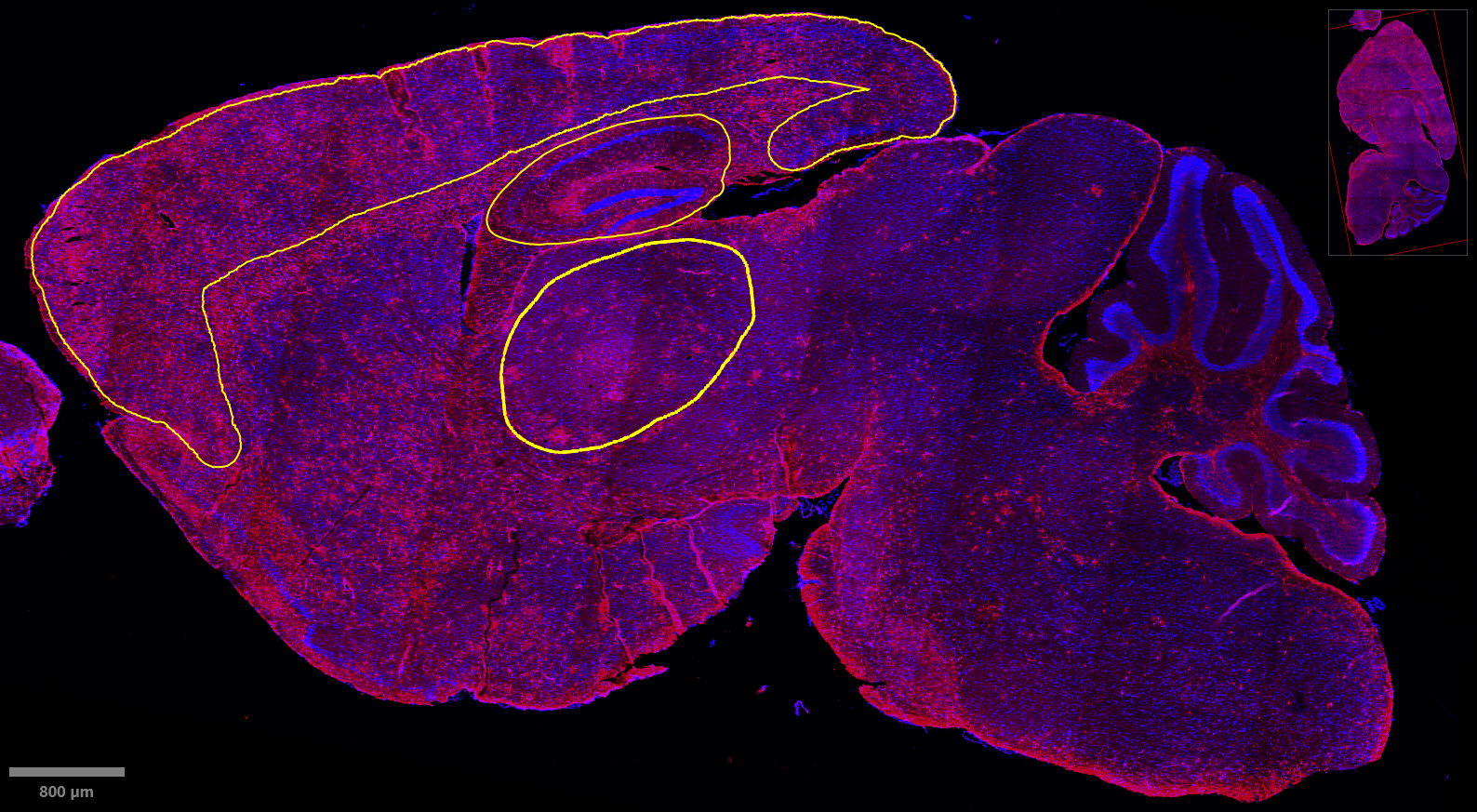

Supplement: Supplementary file 1 [file ijms-27-04994-s001.zip › Supplementary Figure 3 - Representative image of mouse brain sections immunostained for GFAP .tif]

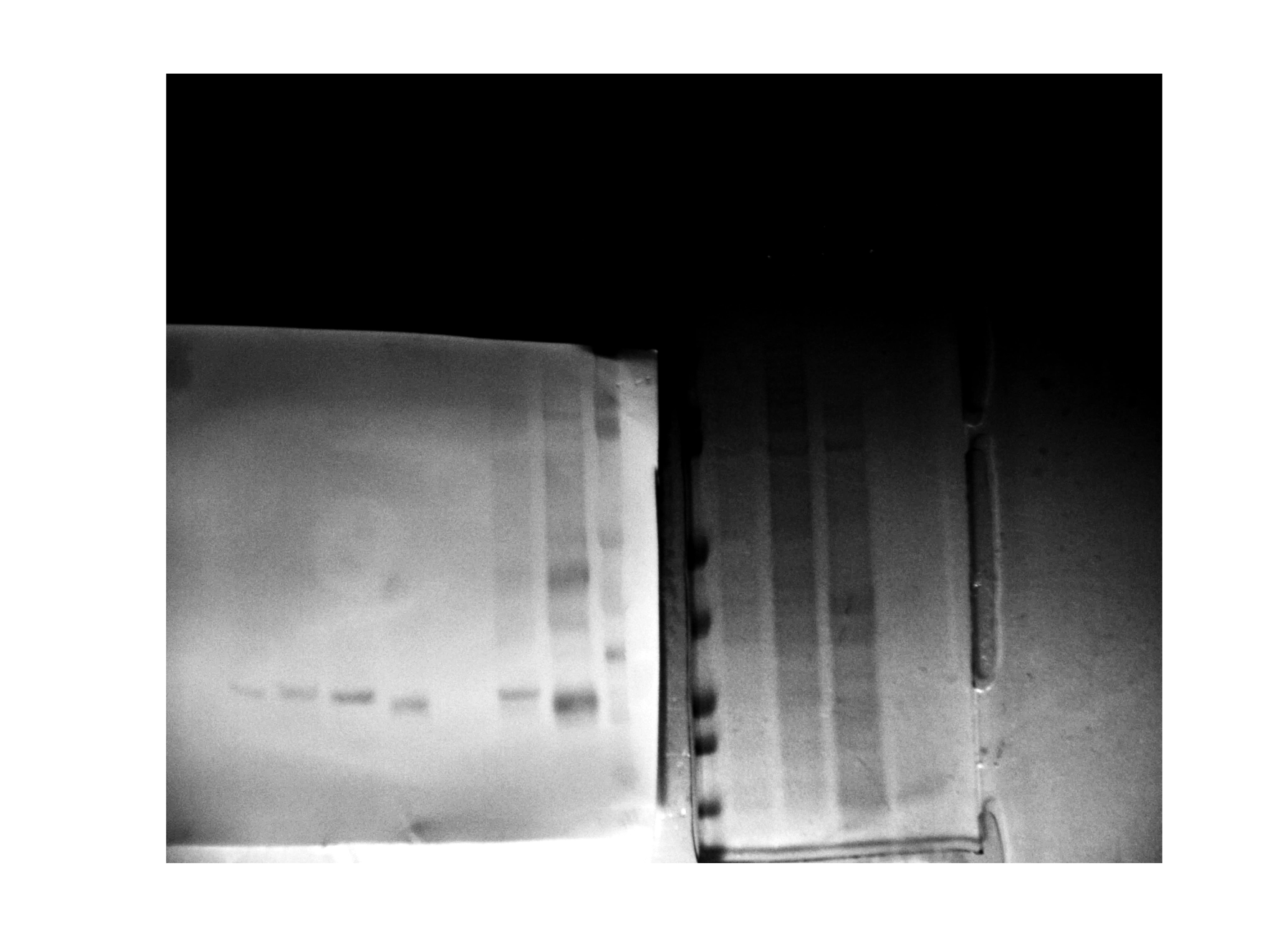

Supplement: Supplementary file 1 [file ijms-27-04994-s001.zip › Supplementary Figure 1A - Western blot - WGA in WME.jpg]

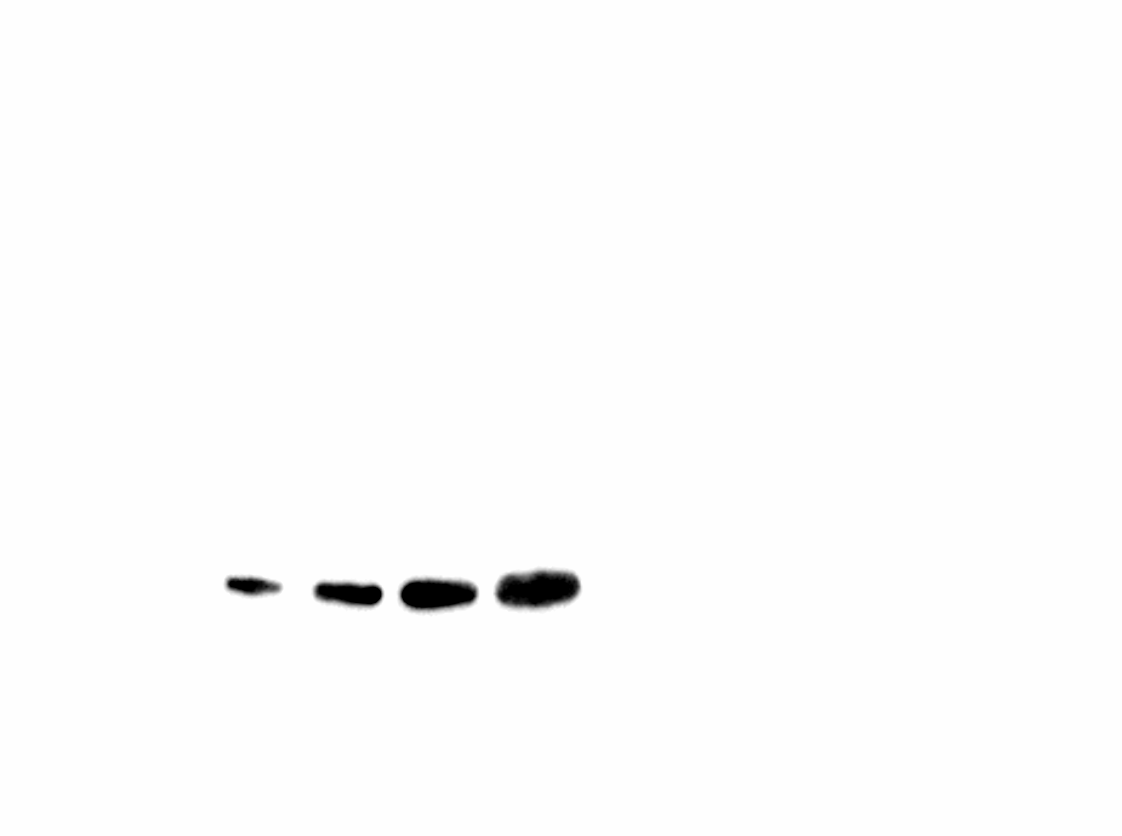

Supplement: Supplementary file 1 [file ijms-27-04994-s001.zip › Supplementary Figure 1B - Standard WGA western blot.tif]

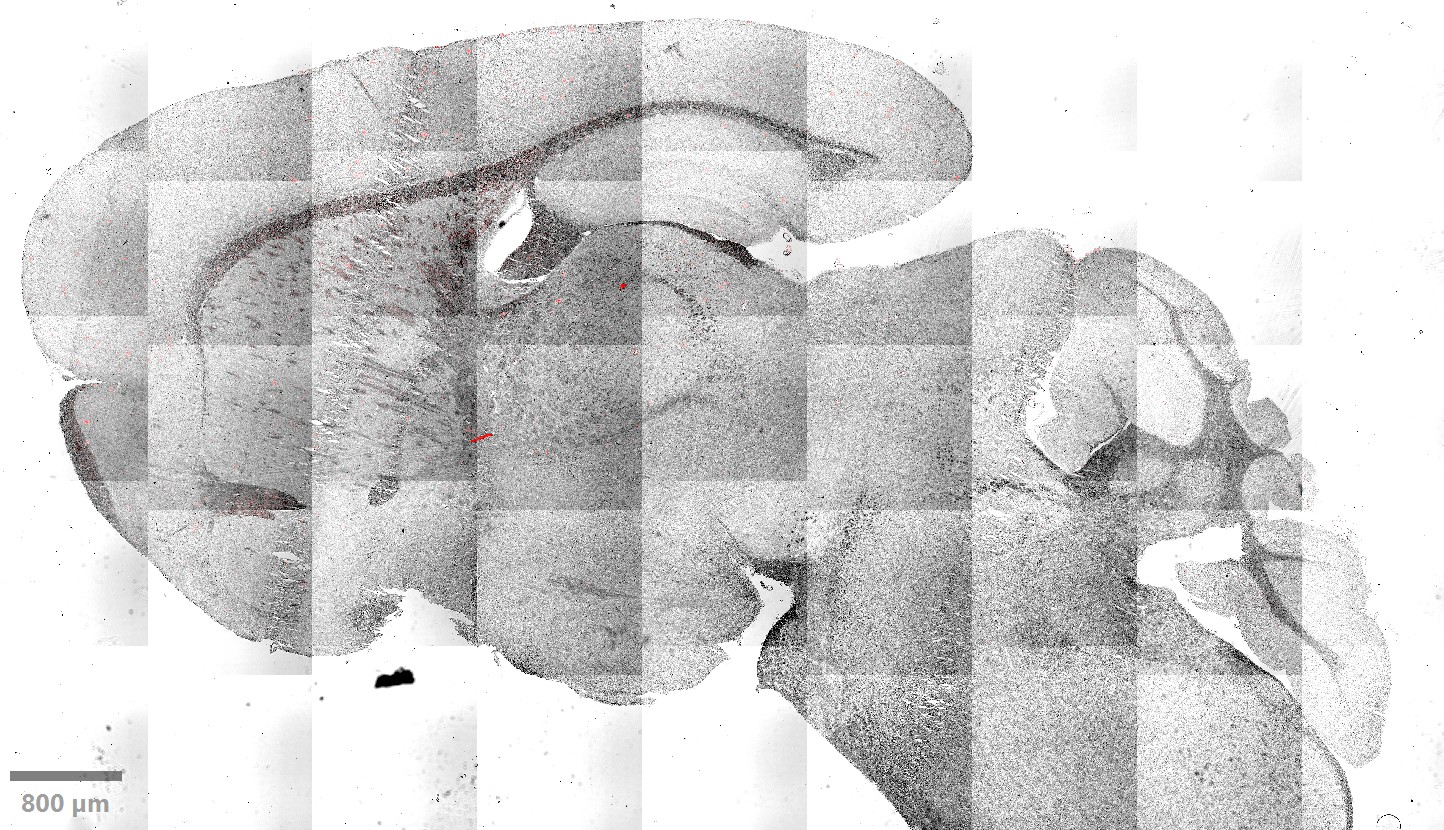

Supplement: Supplementary file 1 [file ijms-27-04994-s001.zip › Supplementary Figure 2A -White for Congo.jpg]

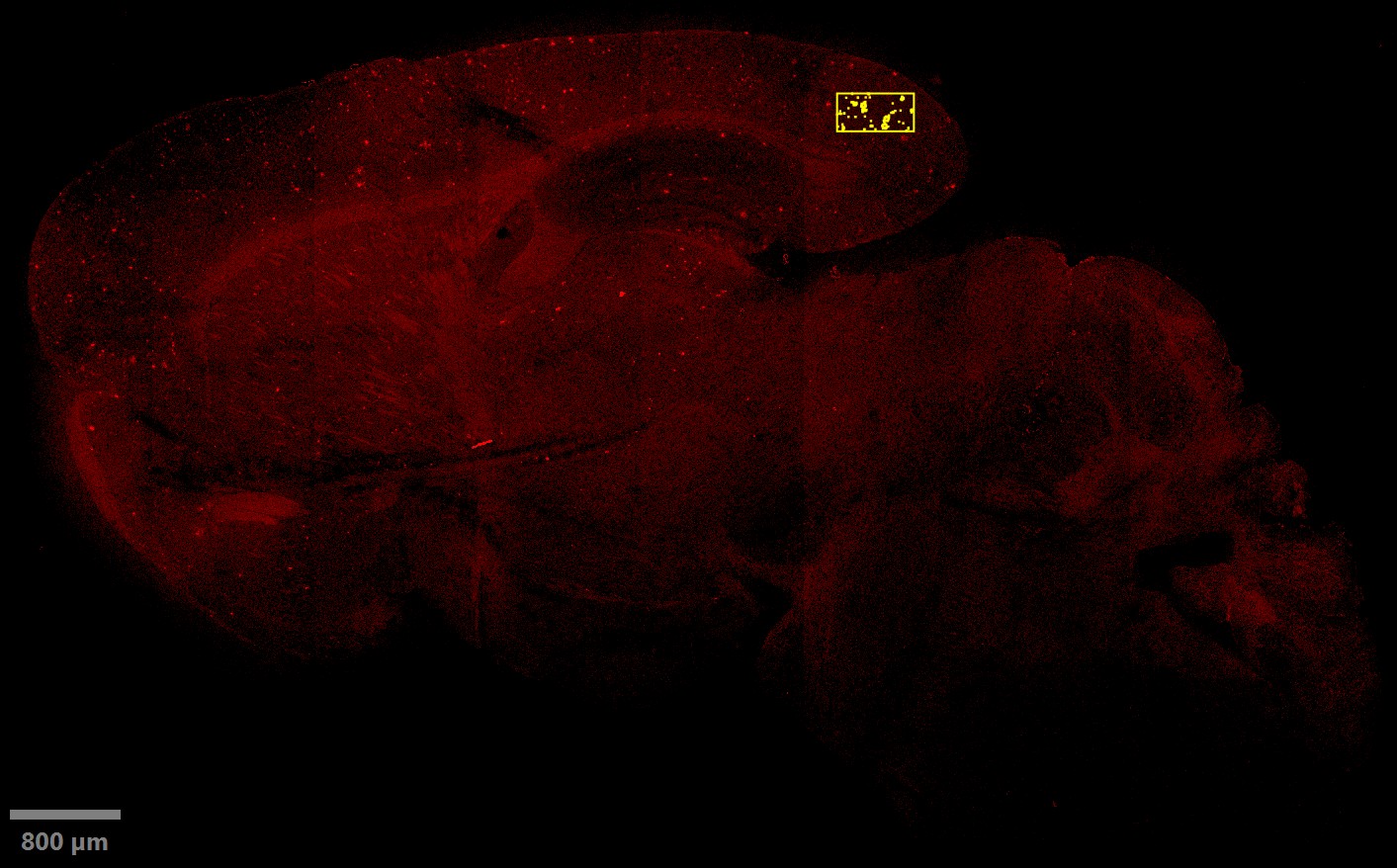

Supplement: Supplementary file 1 [file ijms-27-04994-s001.zip › Supplementary Figure 2B - Congo Red.jpg]
